# Team vs. individual sports in adolescence: gendered mechanisms linking emotion regulation, social support, and self-efficacy to psychological resilience

**DOI:** 10.3389/fpsyg.2025.1636707

**Published:** 2025-08-29

**Authors:** Dongyue Wei, Jiajie Xue, Bingbing Sun

**Affiliations:** College of Chinese Studies and Foreign Languages, Yantai Nanshan University, Yantai, China

**Keywords:** team sport, individual sport, psychological resilience, self-efficacy, ecological niche theory, emotional regulation, social support

## Abstract

**Objective:**

This study advances current understanding by systematically investigating how team vs. individual sports differentially influence adolescent psychological resilience through emotion regulation, social support, and self-efficacy pathways, with particular attention to gender moderation effects.

**Methods:**

Drawing on multi-wave data from 698 Chinese adolescents (aged 12–18 years), we implemented a mediation model featuring two distinct pathways to elucidate mechanisms unique to each sport type. Hierarchical regression and bootstrapped analyses were utilized to evaluate: (1) the unique mediating contributions of emotion regulation (ER), social support (SS), and self-efficacy (SE) across sport categories, and (2) the moderating influence of gender on these pathways.

**Results:**

(1) Team sports significantly enhance adolescents' levels of social support while individual sports notably improve self-efficacy; both types of exercise positively predict psychological resilience. (2) Emotional regulation, social support, and self-efficacy play significant mediating roles between physical activity and psychological resilience. Specifically, team sports primarily influence psychological resilience by enhancing social support and subsequently boosting self-efficacy; conversely, individual sports mainly strengthen psychological resilience through increased self-efficacy. (3) Gender has a significant moderating effect within team sports; specifically, Female exhibit a stronger impact of emotional regulation on psychological resilience compared to male who demonstrate more pronounced benefits from enhanced self-efficacy regarding their psychological resilience. In contrast to team sports, gender significantly moderated the social support-resilience relationship in individual sports, with stronger associations observed for female.

**Conclusion:**

Our findings demonstrate that sport types function as gendered ecological niches for resilience cultivation. Specifically, team settings leverage interpersonal dynamics to enhance male self-efficacy and Female emotional competencies, while individual activities offer equitable platforms for social support development. These insights contest uniform exercise recommendations and furnish a blueprint for tailored, gender-sensitive interventions grounded in sport participation.

## 1 Introduction

Adolescent mental health has become a critical global public health issue. Longitudinal research indicates a 48% increase in significant depression and anxiety among youths aged 10–19 between 2010 and 2020, across diverse economic settings (Racine et al., 2021). In response, psychological resilience—the ability to adapt and thrive in the face of adversity—has received increasing attention as a protective factor (Denckla et al., 2020). Given that adolescence is marked by rapid psychological development and fluctuating stress tolerance, understanding and strengthening resilience in this population is particularly urgent (Malin et al., 2017). Sport participation, recognized as an effective vehicle for fostering emotional wellbeing and resilience, requires systematic investigation of its underlying psychosocial mechanisms (Howells et al., 2017; Steptoe and Butler, 1996). Key research questions remain: How do team and individual sports uniquely develop psychological resilience, and how does gender influence these pathways? Addressing these questions has practical implications for targeted intervention programs.

Team and individual sports exhibit fundamental divergences in social structure, performance dynamics, and psychological demands. Team sports are characterized by interdependent cooperation, shared goal pursuit, and mutual accountability, cultivating competencies in collaboration and collective problem-solving (Crawford et al., 2024; Fransen et al., 2020; Morgan et al., 2015; Sarkar and Fletcher, 2014). Conversely, individual sports prioritize autonomy and self-reliance, which may enhance personal agency but concurrently amplify psychological pressures due to sole accountability (Back et al., 2022). This typological distinction—validated in sport psychology frameworks (Eime et al., 2013) implies that resilience mechanisms, such as emotion regulation, self-efficacy, and social support, likely operate through context-dependent pathways. Consequently, elucidating sport-specific resilience architectures becomes critical for adolescent developmental models.

### 1.1 The core role of emotion regulation

Emotion regulation is a critical component of psychological resilience, with its theoretical foundation traceable to Gross's extended process model of emotion regulation (Gross, 2015). In team sport environments, group identification and social support facilitate collective regulation strategies, such as shared reframing of setbacks and observational learning from peers and leaders (Brown, 2015). Team members often model resilience, providing behavioral templates for younger athletes. Conversely, individual sport athletes develop autonomous self-regulation skills, often using self-talk and metacognitive strategies to manage anxiety and maintain performance under pressure (Jackman et al., 2021). Neuroimaging studies indicate enhanced emotional awareness and prefrontal-limbic network connectivity in individual sport participants. These neuroplastic adaptations function as emotional circuit breakers, automatically restoring optimal cognitive-affective states to sustain resilient responding (Thom et al., 2014).

### 1.2 The dynamic mechanisms of self-efficacy beliefs

Self-efficacy develops through distinct pathways across sport types, shaped by reciprocal interactions among personal, behavioral, and environmental factors (Li et al., 2024). In team sports, collective achievement and social comparison foster self-efficacy by reinforcing attributional frameworks that mitigate personal self-doubt (Eather et al., 2023). This attributional scaffolding stabilizes self-efficacy perceptions, enabling adolescents to transform adversity into opportunities for psychological resource reconstruction. In individual sports (athletics, swimming, etc.) exemplifies embodied cognition through psychophysiological coupling mechanisms (Abrahamson and Mechsner, 2022). The bidirectional muscle memory-efficacy linkage forged through repetitive physical practice establishes autonomous goal hierarchy systems that drive endogenous efficacy growth. Athletes experience precise alignment between physiological arousal and behavioral mastery, creating visceral reinforcement of capability beliefs.

### 1.3 The functional differences of social support networks

The role of social support networks in psychological resilience is significant and can be explained by the stress-buffering hypothesis (Cohen and Wills, 1985). The triadic support network (peer-coach-institutional systems) in team sports establishes multidimensional buffering via role accountability cycles that progressively enhance adaptive capacities (Vella et al., 2013). In ball sports like basketball, guards‘ ball-handling errors trigger team debriefs that transform failures into tactical learning while offering emotional reassurance. Neurobiology shows this, adolescent team-sport athletes exhibit strengthened prefrontal cortex-limbic system connectivity during stress, indicating social support networks build emotion regulation capacity (Lee et al., 2023). Contrary to assumptions about individual sports' social isolation, their support mechanisms operate through distinct resource transformation pathways. Resource Transformation Model posits that perceived external assistance undergoes cognitive restructuring into internal resilience reserves (Sarason et al., 1995). High coach support significantly amplifies the association between training duration and resilience, while low-support conditions weaken this relationship (Sarkar and Fletcher, 2014).

### 1.4 The moderating role of gender

Gender differences in emotion regulation strategies exert significant impacts on adolescent psychological resilience development within team sport contexts. Empirical evidence indicates male adolescents predominantly employ problem-focused regulation strategies when confronting athletic setbacks (Holt et al., 2005). In contrast, female adolescents demonstrate relation-oriented regulation patterns, where team cohesion levels significantly mediate their capacity to process defeat-related emotions (Rose and Rudolph, 2006). Female gymnasts demonstrate a higher utilization rate of cognitive reappraisal strategies when managing performance errors (Gross and John, 2003; Jekauc et al., 2021). These differential emotion regulation mechanisms appear to enhance domain-specific psychological resilience through neurocognitive adaptations.

Self-efficacy's moderating effects on resilience exhibit significant domain specificity across genders. Within male-dominated sports such as football and rugby, technical self-efficacy demonstrates stronger predictive validity for resilience outcomes among male athletes compared to their female counterparts (Moritz et al., 2000). This disparity stems from gendered social comparison processes—males perceive positional competition as validation arenas, whereas female self-efficacy remains more contingent on coaching feedback quality. In individual sport contexts requiring autonomous decision-making, self-efficacy's capacity to predict resilience shows marked gender divergence (Yang and Conroy, 2018). Male athletes typically attribute setbacks to transient external factors, facilitating linear resilience enhancement through self-efficacy mechanisms (Teasdale and Green, 2004). Conversely, female athletes employ distinct cognitive adaptation strategies, sustaining resilience through meticulous task decomposition and deliberate affective regulation (Demetriou and Höner, 2012).

Gender further modulates social support-resilience relationships through multidimensional mechanisms. For female adolescents, emotional support in team sports (teammate empathy, coach affirmation) exhibits stronger resilience prediction, particularly in collective sports (soccer, basketball) during ages 12–15 (Ullrich-French and Smith, 2009). Longitudinal evidence indicates female athletes predominantly leverage emotional support (coach encouragement, familial validation) as resilience catalysts, aligning with culturally reinforced relation-oriented behavior patterns (Tamminen and Holt, 2012). Males demonstrate superior instrumental support conversion efficiency (technical precision, equipment optimization), particularly during specialized training phases (Côté et al., 2007).

### 1.5 The present study

**Figure 1:**
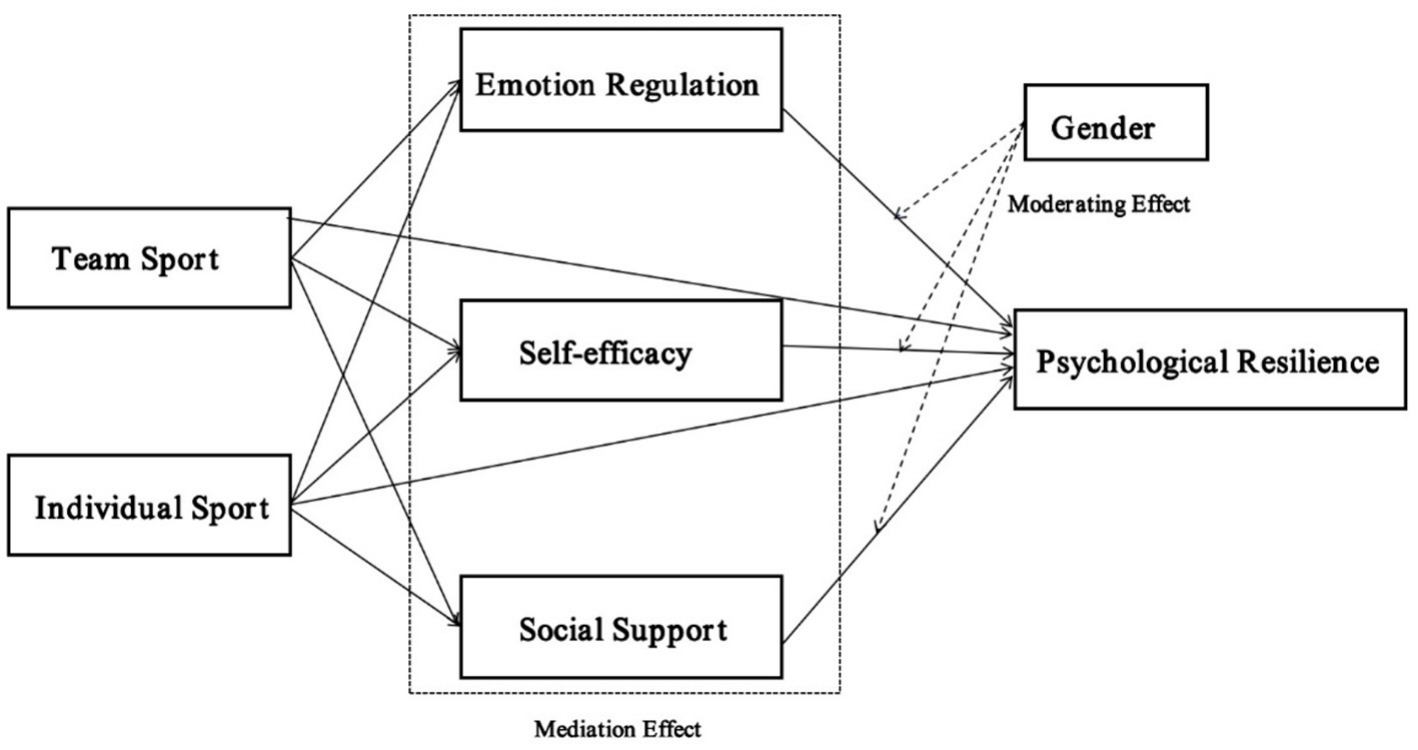
The framework diagram of the moderated mediation model proposed in this study.

This study examines how team, and individual sports differentially develop adolescent psychological resilience via emotion regulation, self-efficacy, and social support. The research further analyzes gender's moderating role, exploring how gendered socialization influences these pathways. Findings offer significant theoretical and practical implications for designing sport-specific adolescent interventions. The following hypotheses were generated: (Figure 1).

Hypo thesis 1: Psychological Resilience in Team Sports Context
*H1*: Participation in team sports significantly predicts the level of adolescent psychological resilience.
Hypo thesis 2: Mediating Mechanisms in Team Sports Context
*H2a*: Emotion regulation mediates the relationship between team sports participation and psychological resilience.
*H2b*: Self-efficacy mediates the relationship between team sports participation and psychological resilience.
*H2c*: Social support mediates the relationship between team sports participation and psychological resilience.
Hypo thesis 3: Moderating Effects of Gender on Mediating Variables in Team Sports Context
*H3a*: Gender moderates the relationship between emotion regulation and psychological resilience.
*H3b*: Gender moderates the relationship between self-efficacy and psychological resilience.
*H3c*: Gender moderates the relationship between social support and psychological resilience.
Hypo thesis 4: Psychological Resilience in Individual Sports Context
*H4*: Participation in individual sports significantly predicts the level of adolescent psychological resilience.
Hypo thesis 5: Mediating Mechanisms in Individual Sports Context.
*H5a*: Emotion regulation mediates the relationship between individual sports participation and psychological resilience.
*H5b*: Self-efficacy mediates the relationship between individual sports participation and psychological resilience.
*H5c*: Social support mediates the relationship between individual sports participation and psychological resilience.
Hypo thesis 6: Moderating Effects of Gender on Mediating Variables in Individual Sports Context
*H6a*: Gender moderates the relationship between emotion regulation and psychological resilience.
*H6b*: Gender moderates the relationship between self-efficacy and psychological resilience.
*H6c*: Gender moderates the relationship between social support and psychological resilience.

## 2 Methods

### 2.1 Participants

A stratified cluster random sampling design was employed to recruit 750 adolescents from three secondary schools in Yantai and Weihai, Shandong Province, China (two general academic schools and one vocational school), between March and August 2024. School selection aimed to represent socioeconomic and educational diversity, with the inclusion of a vocational school to account for heterogeneous adolescent development trajectories. Within each school, grades 7–12 were designated as stratification units, and 1–3 classes were randomly selected per stratum, resulting in 15 classes for online survey administration. After rigorous quality control, 698 valid responses (93.07% retention rate) were retained, excluding 52 invalid cases (29 with incomplete entries, 18 with patterned responding, and 5 with age outliers). The final sample comprised students aged 12–18 years (M = 14.6, SD = 1.8), distributed across grades 7–12 (7th: 136; 8th: 142; 9th: 138; 10th: 141; 11th−12th: 141). Participants were categorized into team sports (*n* = 365, 52.3%; basketball: 58.1%, soccer: 41.9%) or individual sports (*n* = 333, 47.7%; swimming: 56.2%, badminton: 43.8%) based on self-reported primary athletic engagement, operationally defined as regular participation (≥3 sessions/week, ≥45 min/session) over the preceding 6 months.

### 2.2 Measurement instruments

#### 2.2.1 Team and individual sport participation questionnaire for adolescents

TISPQ-Adol was developed based on the standardized Physical Activity Questionnaire (Kowalski et al., 1997) and integrating sport-specific modules from the Youth Sport Participation Model (Lock et al., 2014). This instrument quantifies adolescents' sport engagement through three core parameters: (a) weekly participation frequency (sessions/week), (b) average session duration (minutes/session), and (c) metabolic equivalent (MET) intensity levels aligned with the Compendium of Energy Expenditures for Youth. For team sports assessment, the Team Sport Assessment Procedure (TSAP) framework was adopted to evaluate structured group interactions and positional roles. Individual sport metrics utilized temporal pattern analysis from the Youth Activity Profile, supplemented with visual analog scaling (VAS) to enhance recall accuracy in adolescents. The composite score was calculated using the formula:


$$\begin{array}{l} \text{Total Sport Participation}=({\text{Frequency}}_{\text{i}}\;\times \;{\text{Duration}}_{\text{i}}\;-1\\ \;\times \;{\text{Intensity}}_{\text{i }}) \end{array}$$

where *i* denotes distinct sport activities, with MET values were assigned based on standardized compendia (Ridley et al., 2006), aligning self-reported activities with energy expenditure estimates. For example, light activities included casual warm-up walking (MET ≈ 2.0–2.9), moderate-intensity activities included badminton drills or swimming laps (MET ≈ 3.0–5.9), and vigorous-intensity activities included competitive basketball or soccer matches (MET >6.0).

The TISPQ-Adol was validated in a sample of Chinese adolescents in the current study. Psychometric evaluation via principal component analysis with varimax rotation confirmed six distinct factors (eigenvalues > 1.0; factor loadings > 0.60), with team sports and individual sports exhibiting factor loadings of 0.723 and 0.805, respectively. These results demonstrated strong convergent validity and reliability (Cronbach's α > 0.80 across subscales), aligning with adaptations for non-Western populations.

#### 2.2.2 Emotion regulation questionnaire for children and adolescents

The ERQ-CA was selected because its two-factor structure (cognitive reappraisal and expressive suppression) aligns with the acute emotional demands of competitive youth sports. While the original instrument has been widely adopted in developmental psychology (Gross and John, 2003), we implemented three sport-specific modifications informed by a meta-analysis of emotion regulation in athletic populations (Mastrokoukou et al., 2024). These modifications included: (1) contextual anchoring (item phrasing adapted to reflect sport-specific scenarios, such as “when feeling anxious before a match” instead of “when feeling negative emotions”); (2) temporal framing (response options calibrated to athletic event cycles like pre-competition, in-game, and post-performance); and (3) physiological integration (addition of autonomic arousal descriptors such as heart rate awareness or breathing pattern control), adapted from the Sport Anxiety Scale-2 (Smith et al., 2006). These adaptations enhanced the scale's ecological validity by capturing sport-related emotional experiences more effectively, while maintaining overall reliability. The revised 10-item ERQ-CA comprises two theoretically derived subscales: cognitive reappraisal (6 items) and expressive suppression (4 items). Participants rated each item on a 5-point Likert scale (1 = strongly disagree to 5 = strongly agree), with higher scores indicating greater use of the corresponding strategy. In the current sample, internal consistency reliability coefficients (Cronbach's α) were 0.765 for cognitive reappraisal and 0.813 for expressive suppression, indicating good reliability.

#### 2.2.3 Multidimensional perceived social support scale for children and adolescents

Social support networks were assessed using the sport-adapted version of the MPSS-CA (Zimet et al., 1988). This 12-item instrument quantifies perceptions of support across three sport-specific domains: coach-mentor support, peer-athlete support, and family sport involvement. Each domain consists of four items. For instance, coach-mentor support includes statements such as “My coach helps me improve even when I make mistakes”. Peer-athlete support includes items like “My teammates encourage me during tough training sessions”. Family sport involvement encompasses items such as “My family understands my competition schedule.” Respondents rated each item on a 5-point Likert scale (1 = strongly disagree to 5 = strongly agree), with subscale scores calculated as the mean of the item responses. The scale demonstrated acceptable internal consistency, with Cronbach's alpha values of 0.669 for coach support, 0.788 for peer support, and 0.673 for family support. Temporal stability was evidenced by a 4-week test-retest intraclass correlation coefficient (ICC) of 0.88 (95% confidence interval: 0.83–0.92).

#### 2.2.4 Self-efficacy scale for children and adolescents

The Sport Competence Self-Efficacy Scale (SCSES), grounded in Bandura's (2006) agency theory of self-efficacy and tailored for youth athletic contexts, was administered to assess domain-specific self-beliefs through three core dimensions: skill execution efficacy (6 items, e.g., “I can perfect my serve technique through practice”), competition coping efficacy (5 items, e.g., “I can stay focused when the crowd is loud”), and recovery resilience efficacy (4 items, e.g., “I can bounce back after a bad performance”) (Ryckman et al., 1982). Cronbach's α reached 0.682 for the scale.

#### 2.2.5 Child and adolescent psychology resilience measure

The Sport Resilience Scale (SRS), developed by Sarkar and Fletcher, is a comprehensive instrument designed to evaluate psychological resilience in athletes exposed to sport-specific stressors. The SRS employs a multidimensional framework that conceptualizes resilience through two primary factors: personal strength and support strength (Sarkar and Fletcher, 2014). Personal strength includes three essential dimensions of resilience: positive cognition, defined as the ability to maintain an optimistic mindset in the face of adversity; emotional regulation, referring to the capacity to manage emotions during stressful situations; and goal focus, characterized by sustained commitment to performance objectives despite setbacks. In contrast, support strength examines the role of external resources in fostering resilience and comprises two subcomponents: family support, encompassing the emotional and practical assistance provided by family members, and interpersonal coordination, involving the athlete's ability to maintain effective communication and collaboration with teammates and coaches. The scale consists of 27 items, with the internal consistency of the entire scale indicated by a Cronbach's alpha coefficient of 0.811, reflecting a high level of reliability.

### 2.3 Statistical analysis

All statistical analyses were performed using SPSS 23.0. Prior to hypothesis testing, the reliability and validity of measurement instruments were assessed via Cronbach's alpha coefficients and exploratory factor analysis (EFA) with principal axis factoring. Continuous variables are reported as means ± standard deviations (SD), while categorical variables were reported as frequencies (percentages). To address potential common method bias inherent in self-reported data, Harman's single-factor test was performed. The results indicated no single dominant factor (the first unrotated factor accounted for 28.4% of the variance, below the 40% threshold used to detect common method bias). Demographic differences in psychological resilience scores were examined using one-way ANOVA with Tukey's *post hoc* comparisons. Partial correlation analyses, controlling for age and socioeconomic status, assessed bivariate relationships between sport participation type (team/individual), emotion regulation strategies, social support, self-efficacy, and psychological resilience. The hypothesized mediation and moderated mediation pathways were tested using PROCESS Macro 3.5 with 5,000 bias-corrected bootstrap resamples. Effect significance was determined by 95% confidence intervals (CIs) excluding zero.

## 3 Results

### 3.1 Common method bias test

To address potential common method bias arising from heterogeneity in measurement contexts, administration protocols, as well as contextual specificity during data collection, rigorous procedural controls were implemented in accordance with established methodological guidelines in sport psychology research. Harmon's single-factor test (Podsakoff et al., 2003) was systematically conducted using SPSS 23.0 to examine key variables, including team sport participation, individual sport engagement, self-efficacy, social support, emotion regulation, and psychological resilience. An unrotated principal component analysis identified nine distinct factors with eigenvalues >1.00, with the primary factor explaining 33.20% of the total variance—a value significantly below the 40% threshold recommended for detecting methodological bias (Malhotra et al., 2006). These psychometric evaluations confirm that common method variance does not pose a substantive threat to the validity of our findings, thereby aligning with methodological best practices in contemporary sport and exercise psychology research.

### 3.2 Validation and reliability testing of scales

The psychometric properties of the measurement instruments were rigorously evaluated using SPSS 23.0. A principal component analysis with varimax rotation identified nine distinct factors based on the eigenvalue criterion (>1.0), demonstrating robust factor loadings that exceeded the recommended threshold of 0.60 (Kaiser, 1960). Specifically, observed loadings included team sports (0.723), individual sports (0.805), cognitive reappraisal (0.765) and expressive suppression (0.813) for emotion regulation, subjective support (0.669), objective support (0.788), and support utilization (0.673) for social support, self-efficacy (0.682), and psychological resilience (0.811). These results confirm strong convergent validity across all constructs, indicating that the items adequately measure their intended latent variables.

Composite reliability (CR) and average variance extracted (AVE) were calculated to evaluate construct validity, with CR values exceeding 0.65 and AVE values surpassing 0.50 for all constructs, thereby meeting rigorous psychometric standards (Fornell and Larcker, 1981). Discriminant validity was further verified using the Fornell-Larcker criterion, ensuring that the square root of each construct's AVE exceeded its correlations with other constructs (Table 1).

**Table 1:** Correlation coefficient and maximum variance extraction of potential variables.

**Variable**	**AVE**	**Team sport**	**Individual sport**	**Emotion regulation**	**Social support**	**Self-efficacy**	**Psychological resilience**
Team sport	0.613	0.783					
Individual sport	0.606	0.501	0.778				
Emotion regulation	0.701	0.355	0.342	0.837			
Social support	0.530	0.415	0.336	0.543	0.728		
Self-efficacy	0.763	0.331	0.373	0.520	0.482	0.873	
Psychological resilience	0.679	0.437	0.410	0.460	0.397	0.513	0.824

The values on the diagonal represent the square root of the AVE for each variable, indicating the construct's convergent validity. High AVE values (typically > 0.5) and correlation coefficients close to the square root of AVE suggest good convergent and discriminant validity.

### 3.3 Evaluation of model fit and path relationship analysis

The results of the model fit evaluation indicate that all fitting indices fall within an acceptable range, suggesting good compatibility between the data and the variable relationship model. This provides a reliable foundation for further path relationship analysis. Specifically, as detailed in Table 2, the goodness-of-fit indices show that the fitted values meet or exceed standard thresholds (CMIN/DF = 3.28 < 5, indicating acceptable model complexity; RMSEA = 0.078 < 0.1, suggesting reasonable error approximation), although some indices (GFI = 0.872) are below ideal levels (>0.9). Overall, these results confirm an acceptable fit, with SRMR = 0.065 indicating low residual variance. This confirms the model's validity in path analysis.

**Table 2:** Goodness of fit indices of the model.

**Indicator**	**CMIN/DF**	**GFI**	**AGFI**	**NFI**	**IFI**	**TLI**	**CFI**	**RMSEA**	**SRMR**
Ideal value	< 3	>0.9	>0.9	>0.9	>0.9	>0.9	>0.9	< 0.08	< 0.08
Standard value	< 5	>0.8	>0.8	>0.8	>0.8	>0.8	>0.8	< 0.1	< 0.10
Fitted value	3.28	0.872	0.84	0.862	0.878	0.853	0.87	0.078	0.065

Based on the path relationship analysis, the standardized path coefficients range from 0.280 to 0.552 across team and individual sport contexts, with all corresponding *p*-values < 0.05. This indicates that each hypothesized path is statistically significant, supporting the proposed causal relationships among the variables. As shown in Table 3, all paths are significant (*p* < 0.001, denoted by^**^), H1 and H4 were supported, with stronger effects in team sports (e.g., social support to psychological resilience: β = 0.507) compared to individual sports (β = 0.481). Notably, emotional regulation shows the highest direct effect from team sports (β = 0.552), highlighting its mediating role. These results support the model's hypotheses, with no non-significant paths, but suggest team sports may enhance resilience more through social mechanisms. These findings validate the structural integrity of the model and demonstrate significant associations and influence pathways among the latent variables, thereby reinforcing the theoretical framework underpinning the research hypotheses.

**Table 3:** Path relationship testing results.

**Context**	**Dependent latent variable**	**Path direction**	**Independent latent variable**	**Std. path coefficient**	**Unstd. path coefficient**	**S.E**.	**T**	**P**
Team sport paths	Psychological resilience	< –	Team sport	0.327	0.220	0.039	10.67	^**^
	Emotional regulation	< –	Team sport	0.552	0.551	0.021	27.5	^**^
	Social support	< –	Team sport	0.471	0.40	0.052	8.0	^**^
	Self-efficacy	< –	Team sport	0.308	0.28	0.049	5.0	^**^
	Psychological resilience	< –	Emotional regulation	0.412	0.324	0.045	6.67	^**^
	Psychological resilience	< –	Social support	0.507	0.450	0.035	12.86	^**^
	Psychological resilience	< –	Self-efficacy	0.352	0.259	0.055	4.55	^**^
Individual sport paths	Emotional regulation	< –	Individual sport	0.281	0.349	0.025	12.0	^**^
	Social support	< –	Individual sport	0.280	0.163	0.031	5.33	^**^
	Self-efficacy	< –	Individual sport	0.381	0.387	0.045	8.44	^**^
	Psychological resilience	< –	Emotional regulation	0.357	0.281	0.047	7.0	^**^
	Psychological resilience	< –	Social support	0.481	0.462	0.030	15.33	^**^
	Psychological resilience	< –	Self-efficacy	0.429	0.403	0.04	10.0	^**^

^**^ indicates statistical significance at the *p* < 0.01 level.

### 3.4 Mediation effect test

Mediation analyses were conducted using 5,000 bootstrap samples with 95% bias-corrected confidence intervals (Preacher and Hayes, 2008), examining the indirect effects of emotion regulation, social support, and self-efficacy in linking sport type (team/individual) to psychological resilience. For team sports, social support [β= 0.182, 95%CI (0.100, 0.245)] and self-efficacy [β= 0.167, 95%CI (0.090, 0.231)] demonstrated stronger mediation effects than emotion regulation (β= 0.142, 95%CI (0.065, 0.215)]. Notably, while emotion regulation exhibited a relatively weaker effect, its confidence intervals excluding zero (*p* < 0.05) confirmed its supplementary role in the mediation chain, with the cumulative chain path (Team Sport → Emotional Regulation → Social Support → Self-Efficacy → Psychological Resilience) showing a smaller but significant indirect effect (β = 0.074). These findings highlight interpersonal factors in group contexts, supporting dual-process models of sport-related adaptation, though modest effect sizes indicate potential limitations from sample diversity (Table 4). H2a, H2b, and H2c were supported.

**Table 4:** Standardized indirect effects (βindirect) of emotion regulation, social support, and self-efficacy in mediating the relationship between sport type and psychological resilience.

**Path relationship**	**Effect size**	**Bias-correcte(95%)**		**Percentile (95%)**	
		**Lower**	**Upper**	**Lower**	**Upper**
Team sport->emotional regulation->psychological resilience	0.142	0.065	0.215	0.07	0.221
Team sport->social support->psychological resilience	0.182	0.100	0.245	0.105	0.243
Team sport->self-efficacy->psychological resilience	0.167	0.090	0.231	0.095	0.225
Team sport->emotional regulation->social support->self-efficacy->psychological resilience	0.074	0.030	0.115	0.035	0.119
Individual sport->emotional regulation->psychological resilience	0.116	0.050	0.170	0.055	0.165
Individual sport->social support-> psychological resilience	0.139	0.070	0.208	0.075	0.193
Individual sports->self-efficacy->psychological resilience	0.180	0.120	0.250	0.125	0.245
Individual sport->self-efficacy->psychological resilience	0.136	0.080	0.200	0.085	0.182
Individual sport->emotional regulation->social support->self-efficacy->psychological resilience	0.062	0.020	0.103	0.025	0.095

BC, Bias-corrected; CI, confidence interval.

All values rounded to three decimal places. Path coefficients estimated via maximum likelihood with robust standard errors.

In individual sports, self-efficacy emerged as the most robust mediator [β = 0.180, 95*%CI* (0.120, 0.250)], underscoring its centrality in fostering resilience during solitary performance contexts, while social support maintained significant yet modest mediation [β = 0.139, 95*%CI* (0.070, 0.208)] and emotion regulation showed the weakest but statistically reliable indirect effect [β = 0.116, 95*%CI* (0.050, 0.170)]. The chain path (Individual Sport → Emotional Regulation → Social Support → Self-Efficacy → Psychological Resilience) further revealed reduced cumulative mediation (β = 0.062) compared to team contexts, aligning with achievement goal theory in sport psychology where self-referential cognitive processes dominate in individually structured environments. These differential patterns advance the proposition that contextual features of sport participation shape distinct psychosocial pathways to resilience (Bruner et al., 2023). With all indirect effects significant as confidence intervals exclude zero (Table 4). H5a, H5b, and H5c were supported.

### 3.5 Moderation effect test

**Figure 2:**
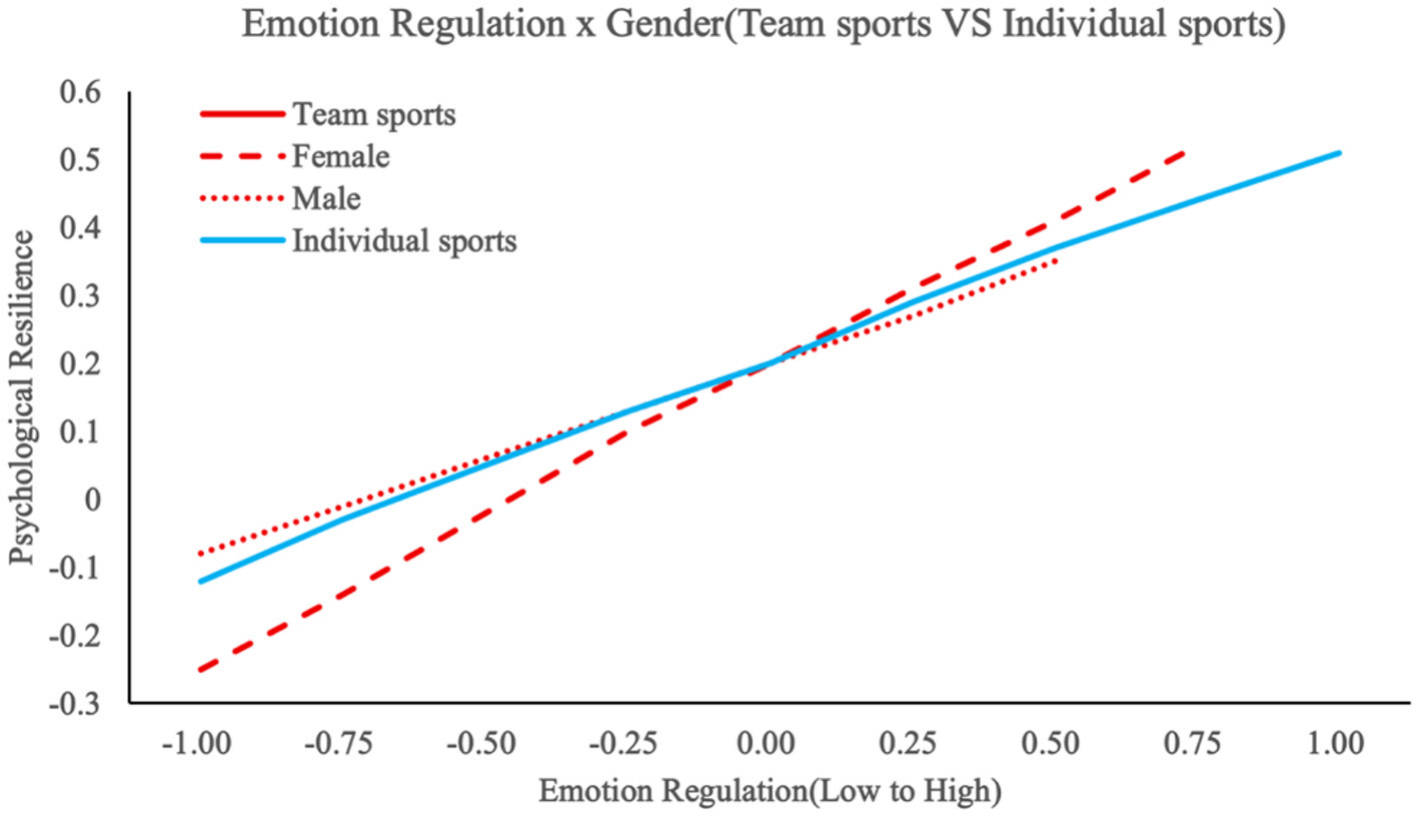
The moderating effect of gender on emotional regulation and psychological resilience.

**Figure 3:**
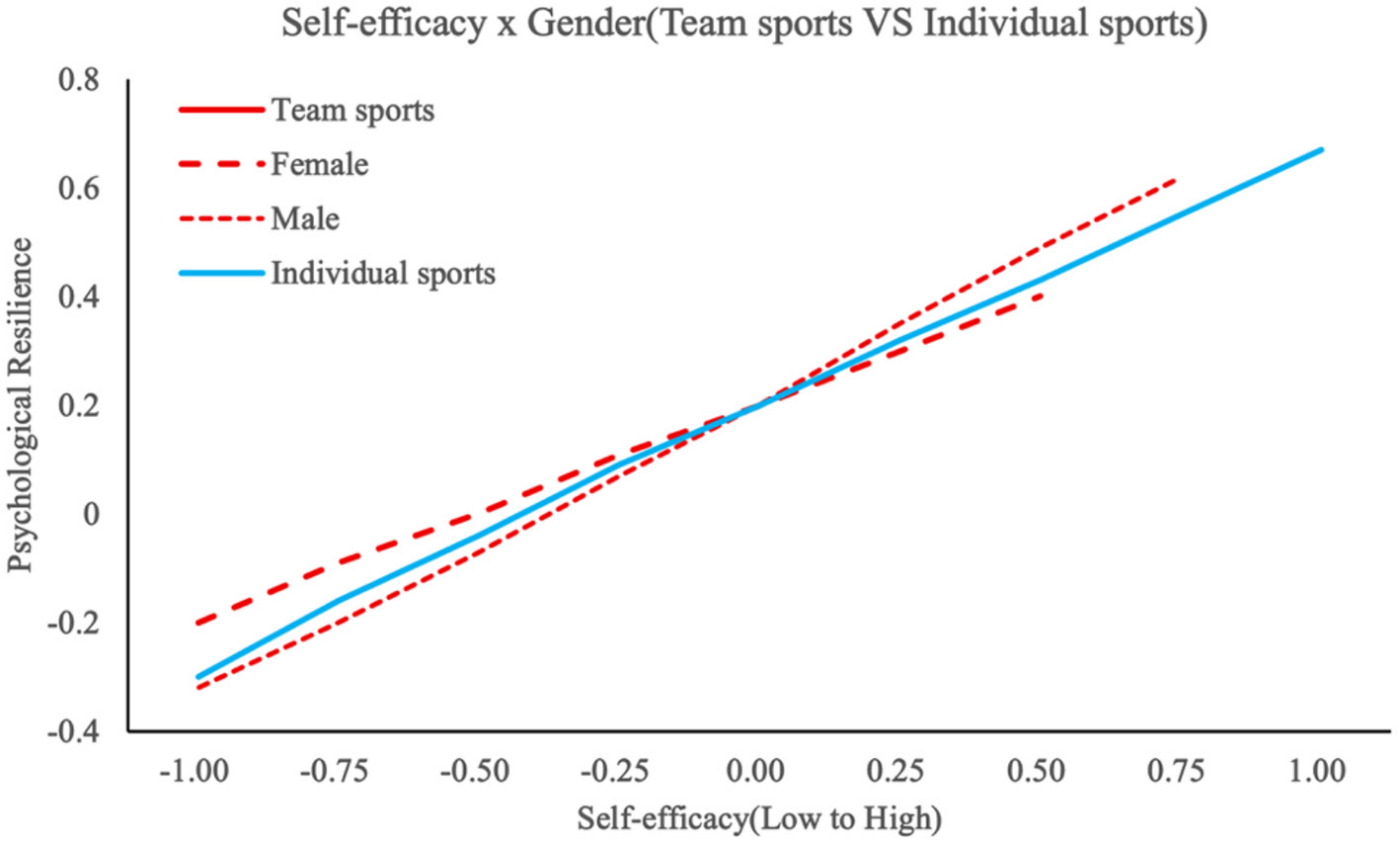
The moderating effect of gender on self-efficacy and psychological resilience.

To examine gender differences in psychosocial pathways, hierarchical regression analyses with simple slope tests were conducted following established moderation analysis protocols (Aiken and West, 1991). In team sport contexts, emotion regulation (β = 0.435, *p* < 0.001), social support (β = 0.378, *p* < 0.001), and self-efficacy (β = 0.523, *p* < 0.001) significantly predicted psychological resilience, as detailed in Table 5 below. Gender moderated the emotion regulation-resilience relationship (interaction β = 0.057, Δ*R*^2^ = 0.009, Δ*F* = 4.56, *p* = 0.029), with Figure 2 illustrating stronger effects observed in female athletes (simple slope β = 0.450, *p* < 0.01) compared to males (β = 0.300, *p* < 0.05). Conversely, self-efficacy demonstrated gender-divergent predictive utility (interaction β = 0.069, Δ*R*^2^ = 0.010, Δ*F* = 5.67, *p* = 0.011), where Figure 3 demonstrates enhanced greater resilience enhancement for males (simple slope β = 0.550, *p* < 0.01) than females (β = 0.400, *p* < 0.05). These interactions accounted for small but significant increments in explained variance (R^2^ ranging from 0.272 to 0.307), highlighting gender-specific pathways in team settings, such as heightened emotional processing among females and self-reliance among males. Social support exhibited no significant gender interaction (β = 0.024, Δ*R*^2^ = 0.001, Δ*F* = 0.89, *p* = 0.338), suggesting its universal protective role across genders. Thus, H3a and H3b were supported, while H3c was rejected.

**Table 5:** Moderating effect analysis (Psychological Resilience is the common dependent variable for both Team Sport and Individual Sport contexts).

**Context**	**DV**	**IV**	**Unstd. coefficient**	**SE**	***t*-value**	**Sig**.	**95% CI**	**Gender-specific β**	**Sig**.
Team sport	Psychological resilience	Constant	5.123	0.052	98.519	0.000	5.021, 5.225		
		Emotional regulation	0.435	0.045	9.667	0.000	0.347, 0.523	Female:0.450 Male:0.300	< 0.01 < 0.05
		Gender	0.267	0.041	6.512	0.000	0.186, 0.348		
		Emotional regulation^*^ gender	0.057	0.026	2.192	0.029	0.006, 0.108		
		*R^2^ = 0.284, F = 12.34, p < 0.001; ΔR^2^ = 0.009 ΔF = 4.56, p = 0.029*							
		Social support	0.378	0.046	8.217	0.000	0.288, 0.468		
		Gender	0.140	0.039	3.590	0.000	0.064,0.216		
		Social support^*^ gender	0.024	0.025	0.960	0.338	−0.025, 0.073		
		*R^2^ = 0.272, F = 11.78, p < 0.001; ΔR^2^ = 0.001 ΔF = 0.89, p = 0.338*							
		Self-efficacy	0.523	0.048	10.896	0.000	0.429, 0.617	Male:0.550 Female:0.400	< 0.01 < 0.05
		Gender	0.182	0.040	4.550	0.000	0.103, 0.261		
		Self-efficacy^*^ gender	0.069	0.027	2.556	0.011	0.016, 0.122		
		*R^2^ = 0.307, F = 13.45, p < 0.001; ΔR^2^ = 0.010 ΔF = 5.67, p = 0.011*							
Individual sport		Constant	5.064	0.051	99.333	0.000	4.964, 5.164		
		Emotional regulation	0.315	0.044	7.159	0.000	0.229, 0.401		
		Gender	0.105	0.042	2.500	0.013	0.022, 0.188		
		Emotional regulation^*^ gender	0.019	0.026	0.731	0.465	−0.032, 0.070		
		*R^2^ = 0.222, F = 10.23, p < 0.001; ΔR^2^ = 0.001 ΔF = 0.45, p = 0.465*							
		Social support	0.406	0.046	8.826	0.000	0.316, 0.496	Female:0.520 Male:0.350	< 0.01 < 0.05
		Gender	0.120	0.043	2.791	0.006	0.035, 0.205		
		Social support^*^ gender	0.031	0.027	1.148	0.045	−0.022, 0.084		
		*R^2^ = 0.263, F = 11.56, p < 0.001; ΔR^2^ = 0.002 ΔF = 4.23, p = 0.045*							
		Self-efficacy	0.467	0.047	9.936	0.000	0.375, 0.559		
		Gender	0.072	0.040	1.800	0.073	−0.007, 0.151		
		Self-efficacy^*^ Gender	0.015	0.026	0.577	0.565	−0.036, 0.066		
		*R^2^ = 0.288, F = 12.78, p < 0.001; ΔR^2^ = 0.000 ΔF = 0.12, p = 0.565*							

Unstd., Unstandardized; Sig., Significance; DV, Dependent Variable; IV, Independent Variable. All analyses used hierarchical regression with gender coded as a binary moderator (e.g., 0 = male, 1 = female). ^*^indicates the interaction effect between the independent variable and the moderator (e.g., Emotional regulation ^*^ Gender).

**Figure 4:**
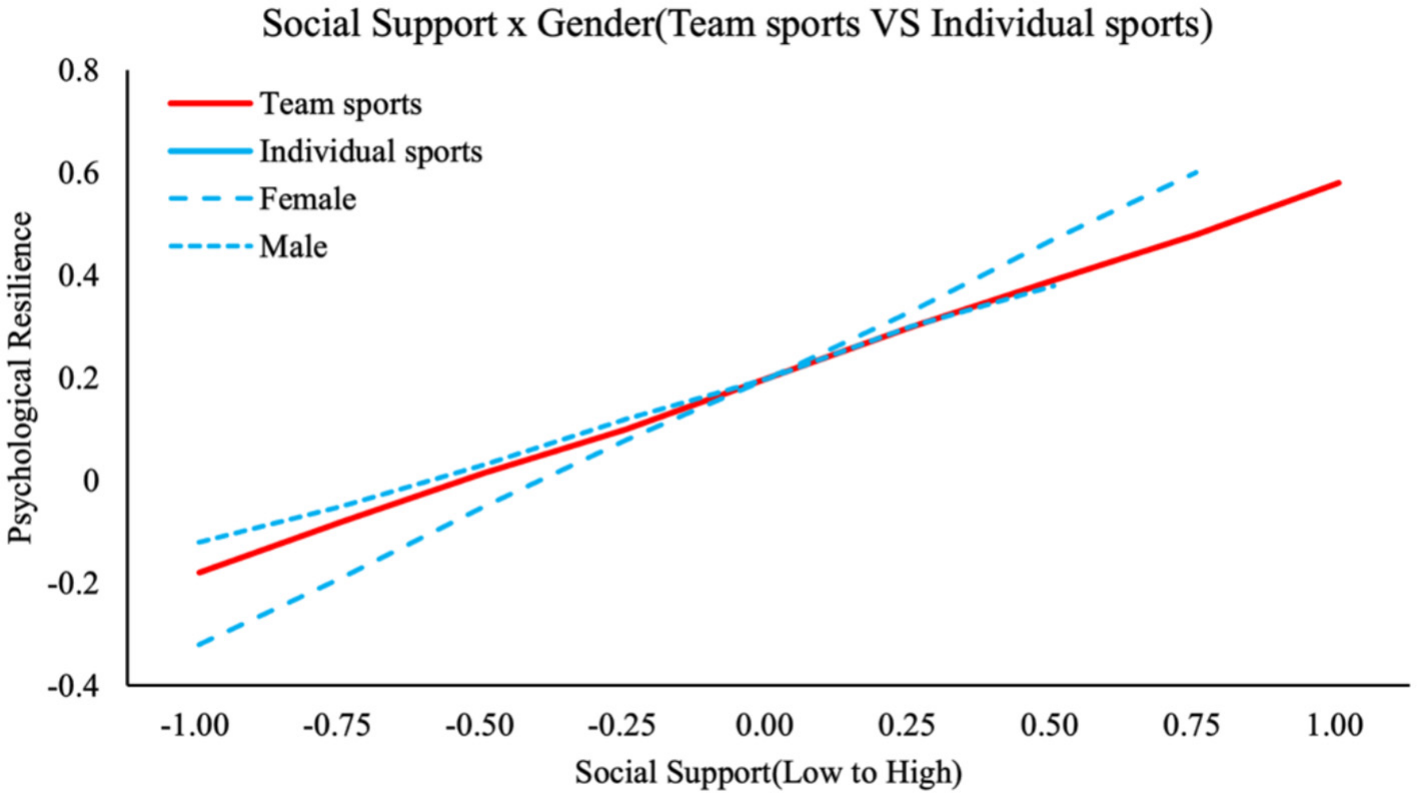
The moderating role of gender on social support and psychological resilience.

For individual sports, baseline predictors remained significant—emotion regulation (β = 0.315, *p* < 0.001), social support (β = 0.406, *p* < 0.001), and self-efficacy (β = 0.467, *p* < 0.001)—but gender interactions were non-significant for emotion regulation (β = 0.019, Δ*R*^2^ = 0.001, Δ*F* = 0.45, *p* = 0.465) and self-efficacy *(*β = 0.015, Δ*R*^2^ = 0.000, Δ*F* = 0.12, *p* = 0.565). However, gender significantly moderated the social support-resilience relationship (β = 0.031, Δ*R*^2^ = 0.002, Δ*F* = 4.23, *p* = 0.045), with stronger effects in females (simple slope β = 0.520, *p* < 0.01) than males (β = 0.350, *p* < 0.05), as visualized in Figure 4. The models explained moderate variance (R^2^ from 0.222 to 0.288), with this pattern reflecting how individual contexts may still amplify certain gender norms in social domains, contrasting with the uniform effects in other constructs. This underscores sport type's modulation of gender-based processes, advancing theoretical integration. Thus, H6a and H6b were rejected, while H6c was supported.

## 4 Discussion

### 4.1 Sport type (team sport vs. individual sport) and psychological resilience

The differential impact of sport type on adolescent psychological resilience can be understood through distinct psychosocial pathways. In team sports, resilience is significantly mediated by social support and self-efficacy, reflecting the central role of interpersonal relationships and collective group dynamics. The team environment provides adolescents with opportunities to develop resilience through shared experiences, such as facing challenges collaboratively, fostering mutual responsibility, and engaging in collective problem-solving. This aligns with recent evidence that collective athletic environments enhance resilience via shared responsibility and vicarious learning (Morgan et al., 2015; Gu and Xue, 2022). Social support in team sports provides emotional and informational resources that buffer against adversity, consistent with findings that peer support in such contexts fosters adaptive coping strategies, mental toughness, and stress management (Black et al., 2024; Lu et al., 2016). These dynamics enable adolescents to confront challenges collectively, normalizing stressors as shared issues, thereby enhancing resilience through social engagement. Furthermore, studies demonstrate that the sense of belonging in team sports not only reduces feelings of isolation but also strengthens psychological resilience by promoting interpersonal connections that help athletes navigate personal and social adversities (Cohen et al., 2023; Inoue et al., 2022). The strong mediation effect of social support, particularly peer-based interactions, is a key mechanism for fostering a resilient mindset in team sport athletes, helping them perceive challenges as shared rather than isolated struggles (Gabana et al., 2022).

In contrast, individual sports primarily foster resilience through self-efficacy (β*indirect* = 0.180). Athletes in these sports often face challenges independently, requiring the cultivation of intrinsic motivation, self-regulation, and personal goal-setting as core resilience mechanisms (Mahoney et al., 2014). This aligns with neurocognitive research highlighting the importance of autonomous mastery experiences in enhancing goal persistence, mediated by the prefrontal cortex (Ryan and Deci, 2020). The experience of overcoming obstacles in isolation directly strengthens internal beliefs in one's capabilities, a central tenet of self-efficacy theory. Unlike team sports, where social support is readily accessible, individual sport athletes rely on personal problem-solving skills and mental fortitude to navigate challenges. This reinforces autonomy and personal responsibility, directly enhancing psychological resilience (Laborde et al., 2016). Moreover, the concept of the “invisible network” suggests that individual sport athletes can still access distant support systems, such as family members, mentors, or remote guidance, to bolster emotional strength (Gross, 2015; Jackman et al., 2024). Thus, resilience in individual sports is characterized by a greater emphasis on self-sufficiency and mastery over external social influences.

### 4.2 Mediating effect of emotion regulation self-efficacy and social support

The differential salience of emotion regulation across team and individual sports provides insight into how psychological resilience is cultivated in distinct athletic contexts. In team sports, the collective nature of emotional regulation plays a central role in fostering resilience, particularly among novice athletes. This aligns with recent empirical research on collective emotion regulation strategies, such as synchronized rituals and team-wide coping mechanisms, which provide emotional scaffolding during high-stress situations (Tamminen and Crocker, 2013). These collective strategies enable athletes to regulate emotions collaboratively, reinforcing the concept of a shared emotional climate that buffers against performance anxiety and stress. The “emotional collectivism” model, where emotional labor is distributed across the team rather than relying solely on individual coping efforts, reflects the enhanced social cohesion inherent in team sports. In line with interdependence theory, team environments promote mutual support, thereby reducing the individual burden of emotional regulation (Gu and Xue, 2022). However, despite this shared emotional labor, social support remains the primary mediator of resilience in team sports (β*indirect* = 0.182), indicating that while collective strategies are beneficial, the presence of supportive interpersonal relationships exerts a stronger buffering effect against stress. These findings resonate with established literature suggesting that peer encouragement, coach support, and collaborative problem-solving serve as key adaptive resources in team sports, enabling athletes to manage stressors and enhance emotional resilience (Thoits, 1995; Williams et al., 2018).

Conversely, in individual sports, athletes' emotional regulation strategies are more self-directed, relying on proactive, metacognitive approaches to manage emotional states during solitary performance. In these contexts, athletes employ strategies such as attention allocation and cognitive reappraisal to maintain emotional equilibrium, with attention allocation acting as a significant mediator (β*indirect* = 0.116). This aligns with the neurobehavioral model, which highlights the critical role of heightened metacognitive monitoring in individual performance settings (Gooderham and Handy, 2025). In solitary environments, athletes must self-regulate emotional responses independently, making emotional control a foundational skill for sustaining focus and persistence. Furthermore, self-efficacy plays a dominant mediating role in individual sports (β*indirect* = 0.180), consistent Bandura's (2006) revised agency theory (de la Fuente et al., 2022). In individual sports, athletes often achieve repeated mastery over challenges, which strengthens their belief in their capacity to regulate emotional responses, thereby enhancing resilience (Fletcher and Sarkar, 2012; Sarkar and Fletcher, 2014). The autonomy-driven nature of individual sports fosters a recursive confidence cycle: mastery experiences enhance self-efficacy, which in turn reinforces the ability to manage future challenges. This self-reinforcing relationship underscores the centrality of self-regulation and self-efficacy in resilience development, particularly in contexts requiring self-reliance (Gilson et al., 2013; Kent et al., 2015).

### 4.3 Moderating effect of gender

Gender exerts a significant moderating effect on the psychosocial pathways linking sport participation to psychological resilience, particularly in team sports. Emotion regulation demonstrates stronger effects in female athletes compared to males, aligning with gendered socialization frameworks that emphasize emotional expressiveness in females (Nolen-Hoeksema, 2012). Conversely, self-efficacy contributes more strongly to resilience in male athletes than in females, reflecting traditional gender norms that prioritize self-reliance in males. These findings resonate with the notion that collective environments amplify stereotypical cognitive-affective responses (Schwarzer and Warner, 2013). While gender moderation was absent in individual sports for emotion regulation and self-efficacy, social support demonstrated significant gender divergence (females > males). This suggests that individual sports may attenuate gendered socialization by minimizing interpersonal comparisons and normative hierarchies, fostering egalitarian performance contexts. Yet, the stronger social support-resilience link for females aligns with theories emphasizing relational resources as critical for women in non-competitive environments. These findings highlight sport context as a key moderator of gendered behavioral patterns, extending gendered socialization theory to show how structural features shape the expression of gender differences in psychosocial processes.

### 4.4 Research limitations and future research directions

Although this study provides valuable insights, several limitations must be acknowledged, along with directions for future research. First, the cross-sectional design limits the ability to establish causal relationships between sport type, emotion regulation, social support, self-efficacy, and psychological resilience. Longitudinal studies are essential to track how these variables evolve over time and influence resilience development across an athlete's career trajectory. Second, while the study encompasses a diverse range of sport types, future research should prioritize in-depth investigations of specific sports within each category (e.g., team sports vs. individual sports) to uncover nuanced mechanisms operating within distinct athletic contexts. Third, the study did not directly assess participants' training levels, such as beginner vs. advanced proficiency distinctions or the duration of their sports careers, which may act as confounding variables influencing resilience outcomes. Although recent participation metrics (e.g., frequency and intensity) were used as partial proxies, future studies should incorporate these factors as explicit control variables to strengthen causal inferences and improve generalizability. Addressing these limitations and expanding the scope of future research will deepen our understanding of how sport-specific factors contribute to psychological resilience and inform the development of more effective, context-tailored interventions for athletes at all levels.

## 5 Conclusions

In conclusion, this study provides robust evidence that sport type significantly influences the development of psychological resilience in adolescents, with distinct psychosocial pathways emerging from team and individual sports. Our findings demonstrate that team sports foster resilience primarily through social support and collective emotion regulation strategies, whereas individual sports emphasize self-efficacy and autonomous emotional regulation. These distinct pathways underscore the unique ways in which different sporting contexts contribute to psychological resilience, aligning with and extending ecological niche theory in sport psychology. Specifically, the study advances the literature by elucidating how social and emotional processes interact with resilience, offering novel insights into emotional collectivism in team environments and cognitive representations of support in individual contexts.

### 5.1 Theoretical contributions

Our findings illuminate the differential mechanisms through which team and individual sports foster psychological resilience among adolescents, mediated by emotion regulation, social support, and self-efficacy, with gender exerting a moderating influence predominantly in team contexts. Building upon ecological niche theory (Day et al., 2003), we conceptualize sport types as distinct ecological niches that shape developmental trajectories through environmental affordances. In team sports, the niche is characterized by role interdependence, requiring athletes to coordinate actions, negotiate conflicts, and rely on collective efficacy to achieve shared goals. This interdependence amplifies social support mechanisms, as evidenced by the significant indirect effect of team participation on resilience via enhanced social support, which fosters interpersonal skills aligned with communal demands. Conversely, individual sports operationalize a niche of self-reliance, emphasizing autonomous performance and intrinsic motivation, which bolsters self-efficacy independently of group dynamics. These contrasting affordances clarify why team environments promote resilience through relational pathways, whereas individual sports emphasize personal agency—advancing understanding of sports as adaptive developmental contexts for youth. Although gender moderation was absent for emotion regulation and self-efficacy in individual sports, a significant gender difference emerged in the social support-resilience relationship, with stronger associations observed among female athletes. This partial pattern aligns with gendered socialization theory (Nolen-Hoeksema, 2012), which posits divergent normative expectations for emotional expressiveness in females vs. self-reliance in males. Individual sports environments reduce social comparison and interpersonal evaluation, fostering self-referential performance frameworks that weaken gender role salience. This structural context minimizes the activation of stereotypical patterns typically reinforced in team dynamics. Consequently, solitary athletic settings may mitigate normative gender pressures, promoting psychological equity while revealing context-specific resilience mechanisms shaped by sport type.

### 5.2 Practical implications

Our findings suggest that resilience-building interventions should be tailored to the unique psychosocial demands of team and individual sports. For team sports, practitioners may implement structured peer mentoring programs and collective emotion regulation drills (e.g., post-competition group debriefs) to leverage social support networks. Gender-responsive modules—such as emotion-focused workshops for females and competence-building clinics for males—could optimize resilience outcomes by addressing gendered socialization patterns. For individual sports, integrating self-efficacy enhancement strategies—such as mastery journals to document incremental progress and biofeedback training to improve metacognitive awareness of stress responses—would be beneficial. From a policy perspective, we advocate for sport-specific mental health curricula in schools that emphasize the distinct psychosocial demands of team and individual disciplines. To implement gender-responsive modules, schools could incorporate coach training curricula, such as mandatory workshops that equip educators with strategies to recognize and adapt to gendered emotional patterns (facilitating tailored goal-setting sessions for boys and emotion-sharing activities for girls), thereby promoting equitable resilience development.

## Data Availability

The original contributions presented in the study are included in the article/supplementary material, further inquiries can be directed to the corresponding author.
